# Risk Factors and Clinical Outcomes of Asymptomatic Bacteriuria in Pregnant Women: A Comprehensive Analysis

**DOI:** 10.7759/cureus.59557

**Published:** 2024-05-02

**Authors:** Debadutta Mishra, Aastha Kalra, Anand R Bhide, Manveer Singh

**Affiliations:** 1 Microbiology, Dr. S. S. Tantia Medical College, Sri Ganganagar, IND; 2 Community Medicine, Dr. S. S. Tantia Medical College, Sri Ganganagar, IND; 3 Community Medicine, Dr. Vasantrao Pawar Medical College, Hospital and Research Center, Nashik, IND

**Keywords:** antenatal care, maternal health, neonatal health, pregnant females, asymptomatic bacteriuria (asbu)

## Abstract

Background

Asymptomatic bacteriuria (ASB) in pregnant women poses risks to maternal and neonatal health. Understanding its prevalence and associated risk factors is crucial for effective management. This study aimed to determine the prevalence of ASB among pregnant women and identify associated risk factors.

Methodology

A cross-sectional study involving 294 pregnant women was conducted. ASB prevalence was determined, and bivariate analysis was performed to identify associated risk factors. Logistic regression analysis was employed to assess the significance of identified risk factors.

Results

The overall prevalence of ASB was 17.34%. Bivariate analysis revealed associations between ASB and maternal age (p > 0.05), socioeconomic status (p < 0.001), previous urinary tract infection (UTI) history (p < 0.001), diabetes mellitus (p = 0.00204), and anemia (p = 0.522). Multivariate logistic regression confirmed significant associations of ASB with maternal age (p = 0.008), parity (p = 0.001), previous UTI (p < 0.001), and diabetes mellitus (p < 0.001).

Conclusion

This study underscores the importance of screening for ASB during prenatal care, particularly among pregnant women with advanced maternal age, higher parity, previous urinary tract infection (UTI) history, and diabetes mellitus. Tailored screening strategies and prompt treatment can mitigate the risks associated with untreated ASB, improving maternal and neonatal outcomes. Healthcare providers should integrate these findings into routine antenatal care protocols to optimize maternal and fetal health.

## Introduction

Asymptomatic bacteriuria (ASB), defined as the occurrence of a substantial bacterial count equal to or greater than 10^5^ colony-forming units (CFUs)/ml in a urine culture, represents a unique clinical entity, particularly due to the absence of any symptomatic urinary tract infections (UTIs) [1]. This condition, while relatively prevalent among the general populace, presents a heightened risk to pregnant women, attributed to the myriad physiological and anatomical transformations inherent to pregnancy. The implications of untreated ASB during pregnancy are far-reaching, encompassing a spectrum of adverse outcomes such as pyelonephritis, preterm birth, low birth weight, and neonatal sepsis. The gravity of these consequences underscores the necessity for a meticulous examination of ASB's prevalence, associated risk factors, and clinical implications to enhance the health outcomes of both mothers and their offspring [2].

The underdiagnosis and undertreatment of ASB in pregnant women complicate its management, signaling a gap in current clinical practices. One of the main reasons could be negligence and lesser and associated taboos related to women's health. A deeper understanding of the pathophysiology of ASB, particularly in the context of pregnancy, is crucial. During pregnancy, physiological changes such as urinary stasis and the dilatation of the ureters, coupled with hormonal influences, create an environment conducive to bacterial growth, thereby increasing the risk of bacteriuria. The progression from asymptomatic to symptomatic bacteriuria, potentially culminating in pyelonephritis, is a significant concern [3]. Pyelonephritis, an acute and severe renal infection, not only endangers maternal health but also poses risks to fetal well-being, including premature delivery and resultant low birth weight leading cause of neonatal morbidity and mortality [2,4].

The identification of risk factors for ASB is imperative for early detection and intervention. Factors such as a history of UTIs, changes in urinary tract anatomy, diabetes, and specific socio-demographic characteristics have been associated with an increased prevalence of ASB in pregnant women [5]. Moreover, the role of screening strategies in managing ASB cannot be overstated. Current guidelines advocate for routine urine cultures during prenatal visits to facilitate the early identification and treatment of ASB, thus mitigating its adverse outcomes [6].

The treatment of ASB in pregnancy is tailored to eradicate the urinary tract bacteria, thereby reducing the risk of symptomatic UTI development and preventing the sequelae of untreated bacteriuria [7-9]. The selection of appropriate antibiotics, with consideration for maternal and fetal safety, is a critical component of management strategies. However, the emergence of antibiotic resistance poses a challenge to the effective treatment of ASB, emphasizing the need for ongoing research into alternative therapeutic options and the judicious use of antibiotics [10].

ASB in pregnancy is a condition of significant clinical relevance due to its association with adverse maternal and neonatal outcomes [5]. The underdiagnosis and undertreatment of this condition highlight the necessity for heightened awareness and proactive management strategies among healthcare providers. A comprehensive approach encompassing the evaluation of prevalence, risk factors, and the implementation of evidence-based screening and treatment protocols is paramount in optimizing maternal and fetal health outcomes. Further research is essential to refine our understanding of ASB, improve diagnostic methodologies, and develop innovative treatment modalities, ultimately enhancing the standard of care for pregnant women worldwide.

The objectives of the study were to determine the prevalence of ASB among pregnant women attending antenatal clinics, identify demographic and clinical risk factors associated with ASB in pregnant women, and find out the clinical implications of ASB in terms of maternal and neonatal outcomes.

## Materials and methods

Study design and setting

The design of the study was cross-sectional. The study population consisted of pregnant women attending antenatal clinics at Dr. S. S. Tantia Medical College and Research Center, Sriganganagar, Rajasthan, between October 2023 and December 2023. Ethics approval was taken prior to the commencement of this study from the Institutional Review Board, which issued an approval letter with reference number IEC/GF/2023/09/03. Consent was obtained or waived by all participants in this study. 

Study participants

The sample size was estimated with the formula [11] for the calculation of sample size for prevalence studies. The formula used was N = 4pq/I^2^, where “p” represents the prevalence of ASB observed in the research conducted by Khapre et al. [5], Z is the critical value corresponding to a 5% type I error rate, and l was the allowable error. Thus, employing the sample size calculations (p = 13.5, Z = 1.96, l = 5), it was determined that the minimum required sample size for the study was 280. A total of 294 women were included in the study, considering nonrespondents.

Data collection and measurement of variables

The data collection process involved the utilization of structured interview schedules conducted during antenatal appointments. Participants included pregnant women who exhibited no symptoms of bacteriuria, had not taken antibiotics within two weeks prior to the urine sample collection date, had refrained from consuming a large volume of water within one hour before sample collection, and did not display any symptoms of sexually transmitted diseases prior to urine collection. Samples for testing were collected using standard operating protocols as per the Indian Council for Medical Research (ICMR) [12]. As per the ICMR standard protocol for urine sample collection, the midstream clean catch method is the most commonly utilized technique for obtaining urine specimens. This approach minimizes contamination by urethral commensals and focuses solely on collecting the midportion of voided urine, which is less likely to be contaminated. When possible, collect the first voided morning specimen because bacteria tend to multiply overnight in the bladder, leading to higher bacterial levels. Since morning collection was impractical for our study participants, urine was collected during the daytime when patients visited the study site, preferably at least four hours after the last void, though bacterial counts may be lower but still significant. It was collected in a sterile, wide-mouth, screw-capped bottle following thorough cleansing of the external genitalia with soap and water, and the same procedure was informed to the patient before handing them the container. No antiseptic was used during this cleansing process to prevent interference with subsequent analysis. Information on demographic characteristics, medical history, urinary symptoms, and urine culture results was also obtained upon these visits.

Data analysis

Descriptive statistics were used to determine the prevalence of ASB. The Chi-square test was used to check the association of socio-demographic variables with ASB. The odds ratio was calculated to check the effect of the factor on the occurrence of ASB. Logistic regression analysis was performed to identify risk factors associated with ASB, adjusting for potential confounders. Clinical implications such as gestational age at diagnosis, maternal complications, and neonatal outcomes were assessed using appropriate statistical methods. A p-value less than 0.05 was considered significant. 

## Results

The overall prevalence of ASB among pregnant women was 51 women, 17.34%. In the bivariate analysis, several risk factors were identified as associated with ASB. Among these, the age of the pregnant women exhibited a significant association, indicating that ASB increased with a lower maternal age. Table 1 depicts the distribution and association of risk factors with ASB in pregnancy among the study participants. Bivariate analysis by age group revealed that participants aged <30 years had higher odds ratios of ASB, compared to those over 30 years (p > 0.05). Gestational age analysis showed no statistically significant associations with ASB, with odds ratios of 1.25 and 1.375 for individuals at <30 weeks and 30-34 weeks, respectively, compared to those at ≥35 weeks. Primigravidas exhibited an odds ratio of 1.42 for ASB compared to multigravidas, yet this association was not statistically significant (p > 0.05). Notably, individuals with lower middle and lower socioeconomic status had significantly higher odds of ASB (odds ratio of 6.74) compared to those of middle class or higher socioeconomic status (reference group), with a significant p-value of <0.001. Previous urinary tract infection (UTI) history strongly associated with ASB, with an odds ratio of 6.07 and a significant p-value of <0.001. Diabetes mellitus and anemia were also significantly associated with ASB, with odds ratios of 3.43 and 1.24, respectively, and p-values of 0.00204 and 0.522, respectively. However, hypertension exhibited an odds ratio of 1.46 for ASB, with a non-significant p-value (p > 0.05).

**Table 1:** Distribution and association of risk factors with asymptomatic bacteriuria in pregnancy among the study participants. ASB: asymptomatic bacteriuria, CI: confidence interval, UTI: urinary tract infection. The data has been represented in numbers (N) with the odds ratio and the 95% confidence interval. *p-value < 0.05 was considered significant.

Risk factor		ASB present	ASB absent	Odds ratio	95% CI	p-value
Age group (years)	20-25	18	71	2.08	1.69-3.01	0.0916
	26-30	24	98	2.01	1.88-2.85	
	>30	9	74	1	-	
Gestational age	<30 weeks	8	35	1.25	0.96-1.75	0.627
	30-34	19	76	1.375	1.11-2.12	
	≥35	24	132	1		
Parity	Primigravida	28	112	1.42	1.20-1.58	0.252
	Multigravida	23	131	-	-	
Socioeconomic status (Modified Kuppuswamy classification)	≥Middle class	12	164	-	-	<0.001
	Lower middle and lower	39	79	6.74	5.93-8.42	
Previous UTI history	Yes	22	27	6.07	1.75-2.41	<0.001
	No	29	216	-	-	
Diabetes mellitus	Yes	11	18	3.43	1.45-2.04	0.00204
	No	40	225	-	-	
Hypertension	Yes	9	31	1.46	1.25-1.67	0.35
	No	42	212	-	-	
Anemia	Yes	15	61	1.24	1.02-1.29	0.522
	No	36	182	-	-	

**Figure 1:**
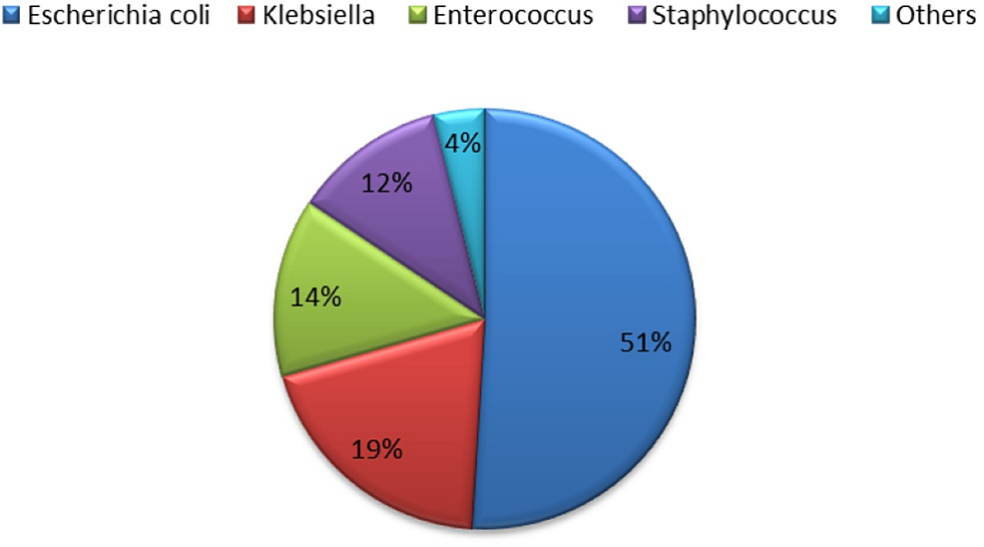
Distribution of bacterial strains in asymptomatic bacteriuria (in percentages).

Figure 1 illustrates a pie chart showing the distribution of bacterial strains identified in urine cultures of pregnant women with asymptomatic bacteriuria (ASB). *Escherichia coli* was the most prevalent strain, followed by Klebsiella, Enterococcus, Staphylococcus, and other lesser common strains.

Nitrofurantoin demonstrated high efficacy across all bacterial strains, with effectiveness ranging from 63.2% to 91.0%. Ciprofloxacin exhibited moderate effectiveness, with efficacy varying between 32.6% and 65.8%. Ampicillin displayed lower efficacy overall, ranging from 3.7% to 48.7%. Trimethoprim-sulfamethoxazole demonstrated moderate to high effectiveness, with efficacy ranging from 43.5% to 78.9%. The same has been depicted in Table 2. 

**Table 2:** Antibiotic sensitivity profile of bacterial isolates (in percentage). The numbers depict the efficacy of the antibiotics in percentages.

Antibiotic	*Escherichia coli* (%)	Klebsiella (%)	Enterococcus (%)	*Staphylococcus saprophyticus* (%)
Nitrofurantoin	87.5	63.2	91.0	78.3
Ciprofloxacin	65.8	42.1	57.9	32.6
Ampicillin	22.4	48.7	14.3	3.7
Trimethoprim-sulfamethoxazole	78.9	55.4	69.2	43.5

**Figure 2:**
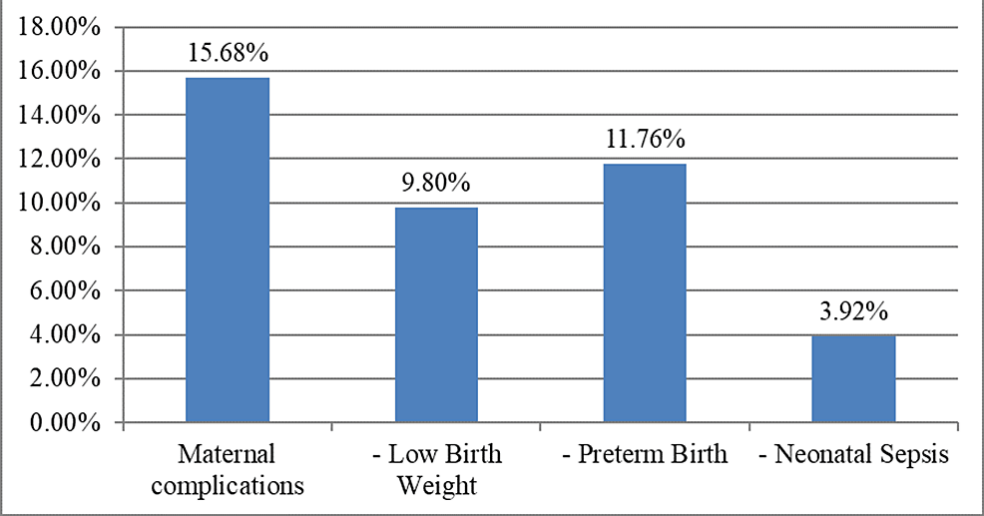
Clinical implications in terms of maternal and neonatal outcomes. The outcomes have been depicted in percentages. The actual values are mentioned in the text for better understanding.

The mean gestational age at diagnosis was 24.6 ± 3.2 weeks. Maternal complications occurred in 8, 15.3% of cases. Neonatal outcomes indicated low birth weight (5, 9.80%), preterm birth (6, 11.76%), and neonatal sepsis (2, 3.92%). These findings underscore the potential impact of untreated ASB on both maternal and neonatal health, as also depicted in Figure 2. 

During the first trimester, the rate of maternal complications was 5, 10.2%, with 4, 7.5% of births resulting in low birth weight, 5, 9.8% in preterm births, and 1, 2.1% in neonatal sepsis. Moving into the second trimester, the incidence of maternal complications increased to 8, 15.8%, with 4, 9.3% of births being low birth weight, 7, 12.6% preterm, and 2, 3.7% affected by neonatal sepsis. By the third trimester, maternal complications reached 20.5%, with 11.6% of births resulting in low birth weight, 8, 15.2% in preterm births, and 2, 4.9% in neonatal sepsis. These outcomes have been tabulated in Table 3. 

**Table 3:** Comparison of clinical implications as per trimester. The data has been represented in percentages. The actual values in numbers have been denoted in brackets.

Trimester	Maternal complications (%)	Low birth weight (%)	Preterm birth (%)	Neonatal sepsis (%)
First trimester	10.2	7.5	9.8	3.7
Second trimester	15.8	9.3	12.6	3.7
Third trimester	20.5	11.6	15.2	4.9

Age demonstrated a statistically significant association with the ASB, with an odds ratio of 1.19 (95% CI: 1.05-1.37, p = 0.008). Parity also showed a significant association, with an odds ratio of 1.31 (95% CI: 1.12-1.52, p = 0.001). Previous urinary tract infections (UTIs) had the highest odds ratio of 1.75 (95% CI: 1.45-2.10, p < 0.001), indicating a substantial association with the ASB. Diabetes mellitus was also significantly associated with odds ratios of 1.48 (95% CI: 1.25-1.80, p < 0.001) and 1.36 (95% CI: 1.18-1.65, p < 0.001), respectively. The same has been tabulated in Table 4. 

**Table 4:** Multivariate logistic regression analysis of risk factors for asymptomatic bacteriuria. *p-value < 0.05 was considered significant. The data has been presented in terms of odds ratio.

Variable	Odds ratio	95% confidence interval	p-value
Age group (years)	1.19	1.05-1.37	0.008
Parity	1.31	1.12-1.52	0.001
Previous urinary tract infection	1.75	1.45-2.10	<0.001
Diabetes mellitus	1.48	1.25-1.80	<0.001

## Discussion

The prevalence of ASB in this study population was found to be 17.34%, similar to another study by Kasinathan et al. [7] and slightly on the higher side from the previous research by Khapre et al. and Gayathree et al [5,8]. Various research studies conducted in India have consistently reported prevalence rates of asymptomatic bacteriuria (ASB) ranging from 6% to 18% [9-13]. Conversely, in developed countries, the prevalence of ASB has been found to be lower, typically falling within the range of 2% to 10% [14]. This disparity in prevalence rates between developed and developing countries underscores potential differences in healthcare practices, sanitation standards, and socioeconomic factors influencing the prevalence of ASB in different regions. The higher prevalence in the present study could be attributed to the study institution being the tertiary care facility attracting referrals.

Similar findings have also been observed in Basumatary et al. [15] and Neelima et al. [16], where 61.9% fell within the age bracket of 18-25 years, and 42.9% were experiencing their second pregnancy (gravida 2). Furthermore, 85.7% of cases were identified during the third trimester of pregnancy. *Escherichia coli* was the most prevalent strain found in this study. Several studies have reported it to be the most common [17,18]. This phenomenon may be attributed to the common occurrence of urinary stasis during pregnancy. Since many strains of *E. coli* thrive in such environments, they are more prone to cause urinary tract infections (UTIs).

Nitrofurantoin and trimethoprim-sulfamethoxazole showed high efficacy across all bacterial strains, similar to the findings of Totadhri et al [18]. Lower sensitivity to other antibiotics can be attributed to empirical use [19]. The results of the logistic regression analysis reveal important associations that can guide targeted screening efforts. Advanced maternal age, low socioeconomic status, higher parity, and a history of previous urinary tract infections emerge as potential risk factors. Additionally, the presence of co-morbid conditions such as diabetes mellitus may contribute to the increased likelihood of ASB during pregnancy.

In line with the present study, Turpin et al. [17] observed that pregnant women with four or more children showed the highest prevalence of asymptomatic significant bacteriuria, and they were also at an earlier stage of gestation. This observation could potentially be attributed to the tendency of women in early pregnancies to seek registration and attend antenatal checkups promptly.

Age-related findings are similar when compared to a study conducted by Laari et al [20]. The incidence of UTI infection was notably higher among pregnant women aged 15-25 years. Conversely, pregnant women aged 26-35 years exhibited a lower likelihood of UTIs compared to those in the 15-25 age group. Furthermore, pregnant women aged 36-45 years were also less susceptible to UTIs compared to their counterparts aged 15-25 years.

In the multivariate analysis, a previous history of UTI emerged as highly significant, along with other co-morbid conditions such as diabetes. The association between UTI and diabetes as predisposing factors was also corroborated by Nguefack et al. [21] and other studies [22,23].

These findings highlight the importance of considering both demographic and clinical factors in risk assessment. Tailoring screening strategies to high-risk groups, such as younger pregnant women or those with a history of UTIs, may enhance the effectiveness of preventive measures. While asymptomatic, untreated ASB can lead to adverse outcomes such as acute pyelonephritis, preterm labor, low birth weight, and even fetal loss as shown by a study conducted in North Indian pregnant women [2].

Similar events were also encountered by the participants of this study, and it was evident that they increased when detected in later trimesters. Therefore, routine screening for ASB during prenatal care is crucial to identify and manage cases promptly. Early detection and treatment of ASB with appropriate antibiotics can reduce the risk of complications, improving maternal and fetal outcomes. Additionally, identifying and addressing underlying risk factors such as diabetes, hypertension, and previous UTI history can further mitigate the likelihood of ASB occurrence during pregnancy. Healthcare providers should emphasize the importance of prenatal visits and adherence to recommended screening and treatment protocols to minimize the impact of ASB on maternal and fetal health.

The analysis of neonatal outcomes provides critical insights into the potential consequences of untreated ASB. Monitoring for neonatal sepsis and other adverse events is essential for comprehensive prenatal care. Incorporating these findings into routine antenatal care protocols can lead to improved outcomes for both mothers and newborns.

Limitations

While the study employed a robust methodological framework for identifying ASB and its risk factors, the limited exploration of certain socioeconomic and lifestyle variables could overlook potential confounders. For instance, dietary habits, stress levels, and access to healthcare services, which could influence the prevalence of ASB and its management, were not accounted for. This oversight might skew the understanding of ASB's risk factors and clinical outcomes. Although this study advances the field's knowledge on ASB in pregnancy, future research could address a few limitations by employing longitudinal designs, enhancing data collection methods, expanding the study population, and incorporating a broader range of socioeconomic and lifestyle variables to provide a more comprehensive understanding of ASB in pregnant women.

## Conclusions

This study underlines the need for targeted screening for asymptomatic bacteriuria (ASB) in pregnant women, considering its observed prevalence and connection with factors such as maternal age, parity, diabetes, and prior UTIs. Implementing risk-specific screening and treatment into prenatal care can substantially improve maternal and neonatal health. The findings advocate for adapting antenatal care protocols to incorporate these risk assessments and treatments, thereby enhancing patient outcomes. Education of healthcare providers on ASB implications can reinforce the benefits of such tailored approaches.
